# T-ARMS PCR genotyping of SNP rs445709131 using thermostable strand displacement polymerase

**DOI:** 10.1186/s13104-018-3236-6

**Published:** 2018-02-15

**Authors:** Rafeeque R. Alyethodi, Umesh Singh, Sushil Kumar, Rani Alex, Rajib Deb, Gyanendra S. Sengar, T. V. Raja, B. Prakash

**Affiliations:** ICAR-Central Institute for Research on Cattle, Grass Farm Road, Meerut Cantt, Meerut, UP 250001 India

**Keywords:** T-ARMS PCR, BLAD, SD polymerase, Taq DNA polymerase, SNP analysis

## Abstract

**Objectives:**

In a recent publication, we reported the successful use of tetra primer-amplification refractory mutation system based polymerase chain reaction (T-ARMS-PCR) for genotyping of rs445709131-SNP responsible for the bovine leukocyte adhesion deficiency (BLAD) in cattle. The SNP is characterized by higher GC content of the surrounding region, hence, the previous protocol utilized dimethyl sulfoxide as PCR enhancer. Here, the reaction cocktail was modified with the use of thermostable strand displacement polymerase (SD polymerase) instead of commonly used Taq DNA Polymerase. The amplification efficiency, reaction sensitivity, specificity, and need of PCR enhancer in reactions containing SD polymerase and Taq polymerase were compared.

**Results:**

T-ARMS-PCR assay is influenced by multiple factors for the correct genotyping necessitating extensive optimization at the initial stages. The described modification enabled generation of all amplicons by 25 cycles whereas the assay with Taq polymerase needed a minimum of 35 cycles. The modified assay amplified all amplicons at a wider range of annealing temperature (50–60 °C), without the addition of dimethyl sulfoxide. The replacement of Taq polymerase with SD polymerase may be beneficial in the T-ARMS assay for development of user-friendly, faster assay which is less affected by the reaction and cyclic conditions.

**Electronic supplementary material:**

The online version of this article (10.1186/s13104-018-3236-6) contains supplementary material, which is available to authorized users.

## Introduction

**Fig. 1:**
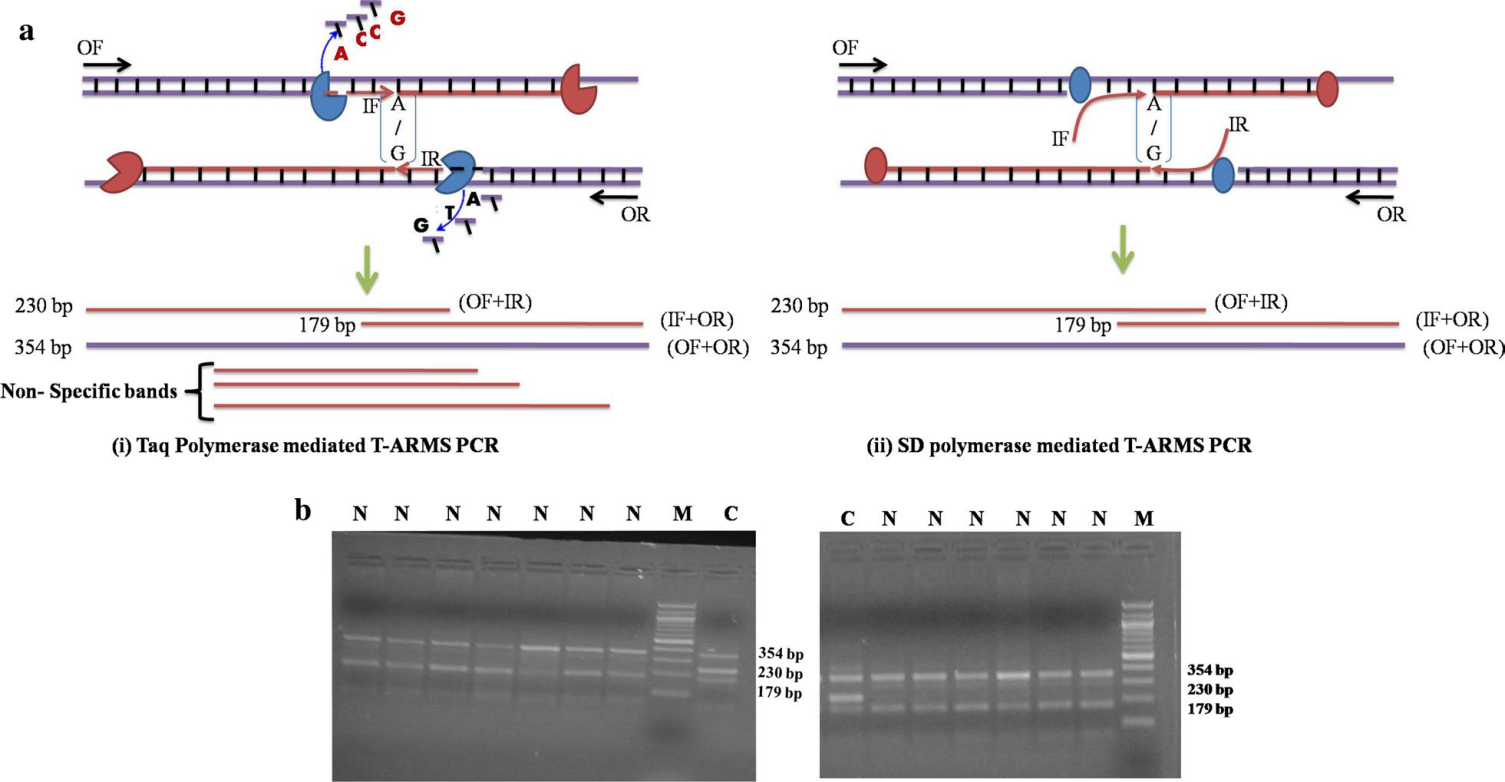
T-ARMS PCR strategy for SNP *rs445709131*. **a** Conceptual diagram of T-ARMS using Taq polymerase (i) and SD polymerase (ii). **b** Genotyping pattern by Taq polymerase (i) and SD polymerase (ii). The outer primers (OF and OR) amplified a 354 bp product. The IF primer generated wild allele with amplicon size of 179 bp while the IR primer generated mutated allele with an amplicon size of 230 bp. *N* wild genotype, *C* carrier genotype, *M* molecular ladder of 100 bp

T-ARMS PCR is flexible, rapid and economical SNP detection tool compared to contemporary genotyping tools such as allele-specific PCR (AS-PCR) [1], high-resolution melting analysis (HRMA) [2], PCR single stranded conformation polymorphism (PCR-SSCP) [3], PCR-primer introduced restriction analysis (PCR-PIRA) [4], real-time PCR-based genotyping [5] etc. It involves a single PCR followed by gel electrophoresis [6, 7]. It utilizes four primers viz. outer forward (OF), outer reverse (OR), inner forward (IF) and inner reverse (IR) primers. The OF/OR primer combination generates the outer fragment of the SNP locus and acts as an internal control for the PCR. The IF/OR and OF/IR primer combinations yield allele-specific amplicons depending on the genotype of the sample used. The inner primers are positioned unequally from the corresponding outer primer to generate amplicons with different sizes and hence easily resolvable in a gel and distinction is made accordingly ([8], Fig. 1). Its use has been reported in disease diagnosis in human [9, 10] and animals including our report on SNP rs445709131 [11]. This rs445709131 is responsible for the bovine leukocyte adhesion deficiency (BLAD) in cattle—an important economical genetic disease of cattle.

In spite of the potential use of T-ARMS PCR in faster, economical, and precise genotyping, the need for extensive standardization at the initial stages hampered its wide application [6, 11]. Also, some sequences [8] and the GC-rich regions are least accessible to this methodology [6]. Identifying the potential of this technology and recognizing its pitfalls, Mesrian Tanha and co-workers [12] modified the outer primers to equalize the primer strengths and included an additional parameter of equal annealing temperature of specific fragments. The use of chimeric primer-based temperature switch PCR (TSP) strategy with T-ARMS primers enabled multiplexing of T-ARMS PCR [13].

Taq DNA polymerase is commonly used in T-ARMS PCR genotyping. The properties of SD polymerase and Taq polymerase are different and influence the T-ARMS PCR differently (Fig. 1a). The Taq DNA polymerase, used in the classical T-ARMS PCR, has 5′–3′ polymerase as well as 5′–3′ exonuclease activity. This 5′–3′ exonuclease activity could degrade the annealed IF and IR primers similar to the 5′ Nuclease Assay [14] by the Taq polymerase of the OF/OR while the 3′ end of IF and IR primers are been extended by another Taq polymerase molecule causing the noisy non-specific bands of varying sizes and intensity (Fig. 1a (i)). The SD polymerase is a variant of Taq polymerase with a 5′–3′ polymerase and 5′–3′ strand displacement activities but devoid of exonuclease activity [15]. The strong displacement activity and lack of exonuclease activity of SD polymerase ensure no degradation of the IF and IR primers hence the chances of non-specific bands is reduced. We hypothesized that the properties of this SD polymerase will be useful for accurate and faster genotyping using T-ARMS PCR. It will be of great use for the faster genotyping and making T-ARMS PCR a preferable genotyping assay without the need of complex, laborious and lengthy standardization steps.

## Main text

### Methods

In T-ARMS PCR, the interaction of primers is a complex phenomenon and may partially depend on its sequence. In order to find the discriminating ability of four primers over all genotypes, it is hence important to have all the genotypes. The current study used a cloned mutant allele (Additional file 1) as mutant genotype.

We have reported the standard procedure of T-ARMS PCR for genotyping of the SNP rs445709131 using Taq polymerase (Sigma-Aldrich, USA, Cat.No.D6677) [11]. The SD polymerase (Bioron GmbH, Germany, Cat. No. 108702) T-ARMS PCR reaction mix was different from the standard T-ARMS (Table 1). In brief, it consisted of 2U of SD polymerase and four primers with outer to the inner primer ratio of 2:1 in a final reaction volume of 25 μl. The reaction condition consisted of pre-heating at 92 °C for 2 min followed by 25 cycles of denaturation at 92 °C for 40 s, annealing at 60 °C for 45 s, extension at 68 °C for 35 s, and a final extension at 68 °C for 5 min. Scoring was done by running the PCR products on a 1.5% agarose gel electrophoresis at 3–5 volts/cm for 40 min. The genotypes are differentiated by checking the amplicon sizes in reference to molecular size markers. The genotyping results are validated by the published method of PCR–RFLP using *Taq1* enzyme (Thermo Fisher Scientific, USA, Cat. No. ER0671) as described [16].

**Table 1:** Optimization of different parameters in SD polymerase based and Taq DNA polymerase-based T-ARMS PCR genotyping

Parameters	SD polymerase based T-ARMS (SNP rs445709131)	Taq DNA polymerase based T-ARMS PCR (SNP rs445709131)
DNA polymerase	2 Units	1 Unit
Outer: inner primer ratio	2:1	1:2
Annealing temperature (°C)	50–60	55
Genomic DNA quantity (ng)	50–100	80–100
Need of PCR enhancers	Not required	DMSO (5%)
MgCl_2_ (mM)	1.5–2	2
No. of PCR cycles	25	35

Each assay is assessed at various PCR cycles viz. 15, 20, 25, 30 and 35 cycles for the generation of all amplicons of sufficient intensity. Assessment is made by visualization on a 1.5% agarose gel. The correct genotyping ability of each protocol without non-specific band formation is also assessed by visualization on a 1.5% agarose gel. Temperature sensitivity of each protocol is measured by gradient PCR from 50 to 60 °C with an increment of 2 °C. DMSO used as PCR enhancer in the Taq polymerase based genotyping assay at a five percentage optimized level [16].

### Results and discussion

**Fig. 2:**
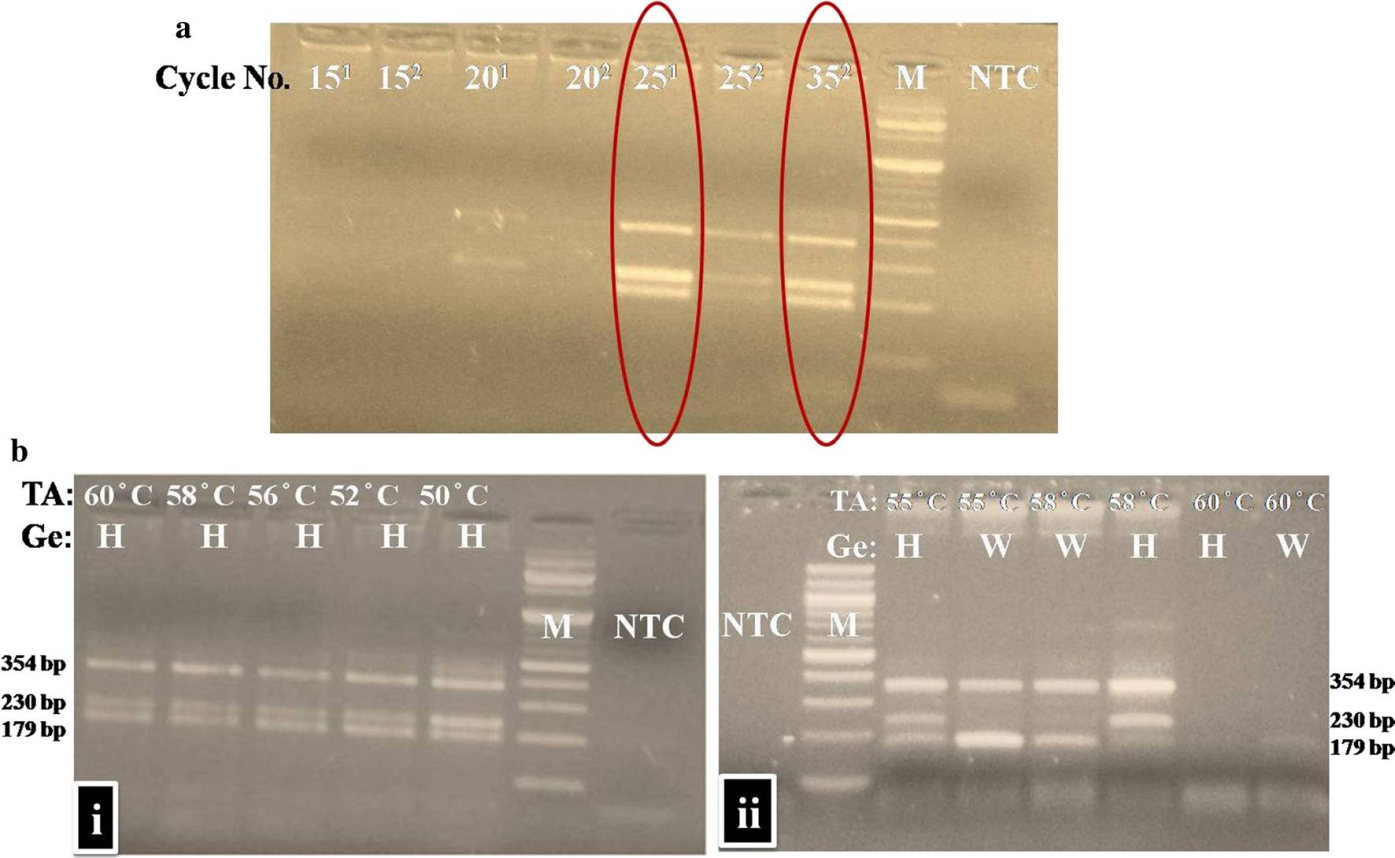
Comparison of SD polymerase and Taq DNA polymerase with respect to amplification efficiency (**a**) and influence of annealing temperature (**b**). **a** Superscript 1: T-ARMS PCR with SD polymerase. Superscript 2: T-ARMS PCR with Taq DNA polymerase. *M* molecular ladder of 100 bp, *NTC* no template control. **b** (i) SD polymerase at various TA on heterozygous (H) genotype (ii) Taq DNA Polymerase at various TA on heterozygous (H) and Wild (W) genotypes (Ge) in presence of DMSO (5%). *M* 100 bp ladder, *NTC* non-template control

The T-ARMS genotyping using SD polymerase generated good amplification from 25 cycles onwards and at all the tested temperature range of 50–60 °C without need of DMSO (Fig. 2) while the T-ARMS genotyping using Taq DNA polymerase generated all the expected amplicons at 35 cycles of PCR and at a narrow temperature of 55 °C in the presence of five percentage DMSO.

T-ARMS PCR using both the Taq DNA polymerase and SD polymerase generated expected genotype pattern, hence have similar specificity; normal genotype for rs445709131 showed a 354 and 179 bp amplicons while the carriers showed an additional band of 230 bp (Fig. 1b). The mutant allele showed an expected genotyping pattern of only 354 and 230 bp amplicons indicating the discretionary ability of the primers on all genotypes (Additional file 1: Figure S2).

A higher concentration of outer primers found beneficial in case of SD Polymerase. It may be due to the fact that the outer primers are required for both outer–outer fragment generation as well as outer–inner fragment generation. In the case of Taq DNA polymerase, the 5′–3′ exonuclease activity may degrade the inner attached primers [14]. Hence, it is sometimes needed to add more inner primers compared to outer primers [17]. This property of Taq DNA polymerase may cause non-specific band formation [11]. The optimum required unit of SD polymerase is tested for the amplification of all the amplicons. While 1U of SD polymerase generated low-intense outer amplicons, use of 2U of SD polymerase gave good amplification of both outer and inner amplicons (Additional file 1: Figure S3). In the case of Taq Polymerase, increasing the quantity of enzyme from 1U to 2U didn’t show any beneficial effect in T-ARMS PCR genotyping (Additional file 1: Figure S3). A comparison of the use of Taq polymerase and SD polymerase in polymerase chain displacement reaction (PCDR) reported a significant higher efficiency of SD polymerase while Taq polymerase fails to give successful pattern in four and six primer PCDR [15]. It might be due to the fact that complex PCR with Taq polymerase need more optimization for the correct genotype pattern as shown in case of T-ARMS PCR for the rs445709131 [11].

The T-ARMS genotyping using SD polymerase generated good amplification at 25 cycles, making this protocol faster than Taq Polymerase-based genotyping which otherwise required 35 cycles to generate visible gel resolvable amplicons (Fig. 2a). Similarly, the higher efficiency of SD polymerase was reported in the case of long-range PCR and loop-mediated isothermal amplification (LAMP) [15]. The strong displacement activity of SD polymerases like Bst polymerase and its high thermostability like Taq DNA polymerase enabled the SD polymerase a suitable polymerase in complex PCR like T-ARMS PCR.

The role of annealing temperature in T-ARMS PCR is reported [6, 11]. The SD polymerase showed good amplification in annealing temperatures varied from 50 to 60 °C (Fig. 2b (i)) whereas the Taq polymerase gave amplification of all amplicons only at 55 °C with optimized conditions of DMSO (5%) and MgCl_2_ (2 mM) (Fig. 2b (ii)). This higher susceptibility of Taq polymerase necessitates finding of the right annealing temperature with a right combination of MgCl2 and DMSO. It may indicate that Taq polymerase based T-ARMS PCR is sensitive to the changes in annealing temperature and reaction conditions which may be one of the reasons why T-ARMS PCR needed higher initial standardization as reported [6].

Modified assay with SD polymerase successfully generated correct genotype pattern with absences of DMSO while it was needed in case Taq DNA polymerase for genotyping of rs445709131 (Fig. 1b). The higher GC content of the region surrounding the rs445709131 (60.3%) or the difference in the GC content of the primers viz. OF (53.6%), OR (53.6%), IF (61.9%), IR (51.7%) necessitate the need of addition of DMSO in the reaction mix with Taq polymerase [11]. The properties of DMSO is useful in bringing down the melting temperature of the primers [18, 19], hence important for the amplification moderate to high GC rich regions [11]. The strong displacement activities of SD polymerase lead to better amplification of GC-rich regions [15], hence PCR enhancer like DMSO is not added to the reaction mix with SD polymerase. Hence, use of SD polymerase can generate amplicons in 25 PCR cycles in broad range of annealing temperature (50–60 °C) without need of addition of PCR enhancer like DMSO while the Taq Polymerase based T-ARMS required 35 cycles, needed DMSO addition and amplified in a specific narrow range of annealing temperature (55 °C).

### Conclusion

SD polymerase based T-ARMS PCR genotyping is faster as the assay can be completed in 25 cycles compared to that of Taq polymerase based assay. It is less affected by the annealing temperature and reaction mix. The specificity of T-ARMS with SD Polymerase is same or better than the Taq DNA polymerase. In order to generate resolvable amplicons using Taq DNA polymerase extensive standardization with respect to the annealing temperature, salt (MgCl_2_) concentration, DMSO was needed. The SD polymerase, on the other hand, generated a T-ARMS genotyping pattern with less optimization. Hence, replacing the Taq DNA polymerase with SD polymerase can complete the T-ARMS PCR faster. The broader annealing temperature and lack of DMSO usage in SD polymerase may reduce the need for extensive standardization and may give better genotyping of GC-rich regions.

## Limitations

This study demonstrates the use of SD polymerase over Taq polymerase in T-ARMS PCR based genotyping of a single SNP. Hence, concluding the use of SD polymerase in T-ARMS PCR needs validation on more numbers of SNP. If can be validated, the major drawback of the T-ARMS PCR- extensive standardization and lack of GC rich amplification—can be overcome making the T-ARMS assay very user-friendly.

## Additional file

**Additional file 1.** Generation of Mutant genotypes/Genotyping of mutant using PCR–RFLP and T-ARMS PCR/Effect of use of 1 and 2 units of SD and Taq polymerase.

## Abbreviations

T-ARMS PCR: tetra primer-amplification refractory mutation system-PCR
SD Pol: strand displacement polymerase
DMSO: dimethyl sulfoxide
AS-PCR: allele specific-PCR
HRMA: high-resolution melting analysis
SSCP: single strand conformation polymorphism
PIRA-PCR: primer induced restriction analysis-PCR
OF: outer forward
OR: outer reverse
IF: inner forward
IR: inner reverse
BLAD: bovine leukocyte adhesion deficiency
