# Supplementary material for: T-ARMS PCR genotyping of SNP rs445709131 using thermostable strand displacement polymerase
Source: BMC Res Notes. 2018 Feb 15;11:132. doi: 10.1186/s13104-018-3236-6 (PMC5815177; doi:10.1186/s13104-018-3236-6)
Supplement: Supplementary file 1 — Additional file 1. Generation of Mutant genotypes/Genotyping of mutant using PCR–RFLP and T-ARMS PCR/Effect of use of 1 and 2 units of SD and Taq polymerase. [file 13104_2018_3236_MOESM1_ESM.docx]

**Method**

*Generation of mutant genotype*

Bull calves (300) were screened for SNP rs445709131using standard PCR-RFLP. Screened animals showed the presence of wild homozygous and heterozygous genotypes in the population. Hence, the mutant genotype is created (SupplFig 1) as follows; the PCR-RFLP of heterozygous genotype was carried out using *Taq1*restriction enzyme(Thermo Fisher Scientific, USA, Cat.No. ER0671)and yielded both normal and mutated allele on the agarose gel. The mutant allele leaves a 354 bp undigested fragment which was gel eluted using GenElute™ Gel Extraction Kit (Sigma-Aldrich, USA, Cat.No.NA1111) and cloned into pTZ57R/T vector. The plasmid was isolated using GenElute™ Plasmid Miniprep Kit (Sigma-Aldrich, USA, Cat.No.PLN70) and digested by *Taq 1*to ensure correct cloning (Suppl Fig 2).


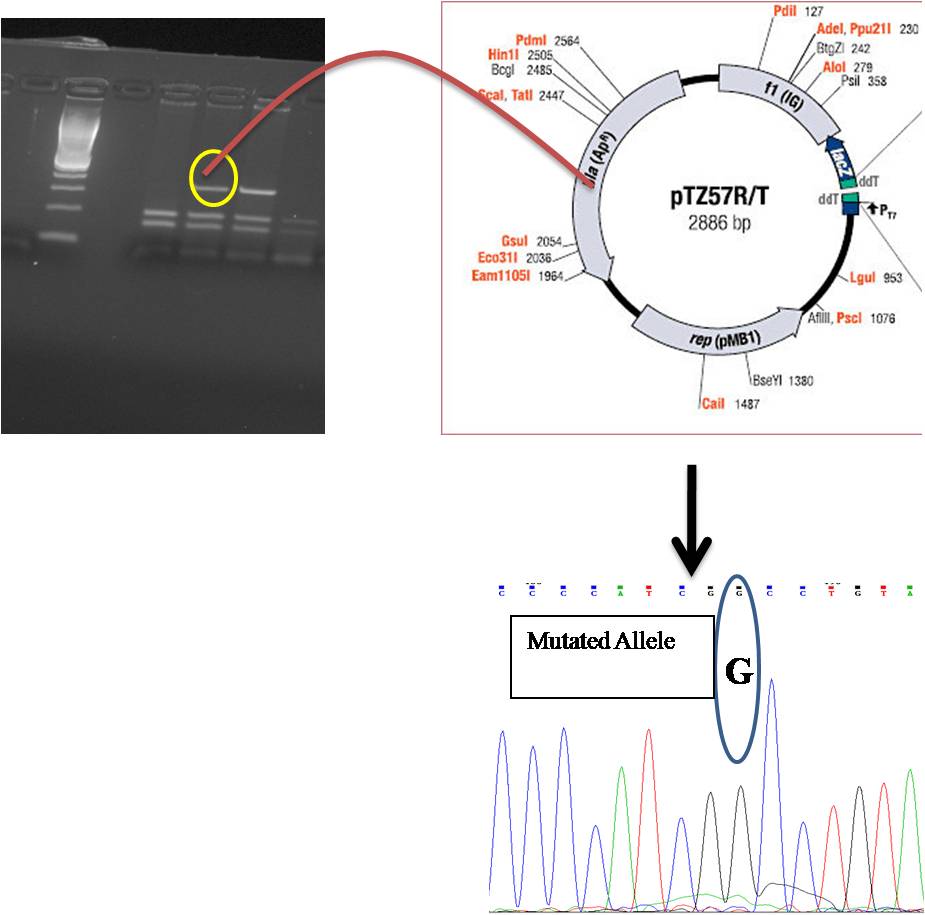


Fig 1. Generation of mutant genotype.

**Results**


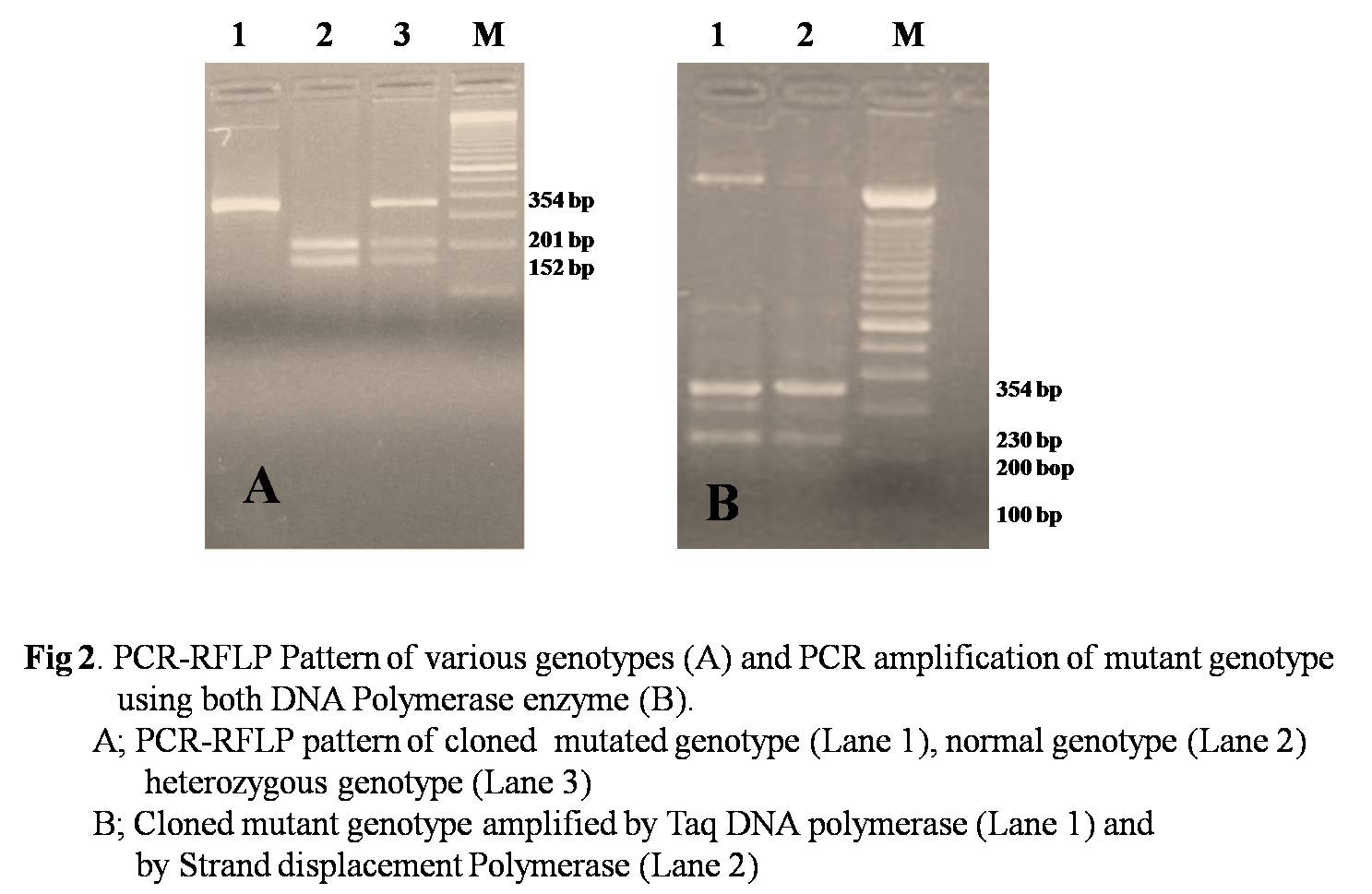
**Fig 2.** PCR-RFLP Pattern of various genotypes (**A**), T-ARMS PCR amplification of mutant genotype using both the DNA polymerase (**B)**.

**(A);** PCR-RFLP pattern of cloned mutated genotype (Lane 1), Wild genotype (Lane 2) and Heterozygous genotype (Lane 3)

**(B);** Mutant genotype amplified by the Taq DNA polymerase (Lane 1) and the SD polymerase (Lane 2). M (100 bp Molecular marker)

The mutant genotype showed only 354 bp and 230 bp bands in T-ARMS PCR (Fig 2). A weak non-specific band was noticed in the case of Taq Polymerase added T-ARMS which was absent in SD Polymerase. It may be due to the difference in the protocols of plasmid DNA extraction compared to genomic DNA extraction. Plasmid was extracted by GenElute™ Gel Extraction Kit while genomic DNA was extracted by the standard phenol-chloroform method. It is in concordance with the report that the difference in the DNA extraction protocol leads to different reproducibility in the results in T-ARMS PCR using the same primer [6]. Moreover, the specificity depends on the primer sequences and the primer interactions which vary depending on the salt, pH, other factors in the reaction mix. The SD polymerase was least influenced by the extraction procedure as it is evident by the absence of non-specific bands with the plasmid DNA extracted by the GenElute™ Gel Extraction Kit.


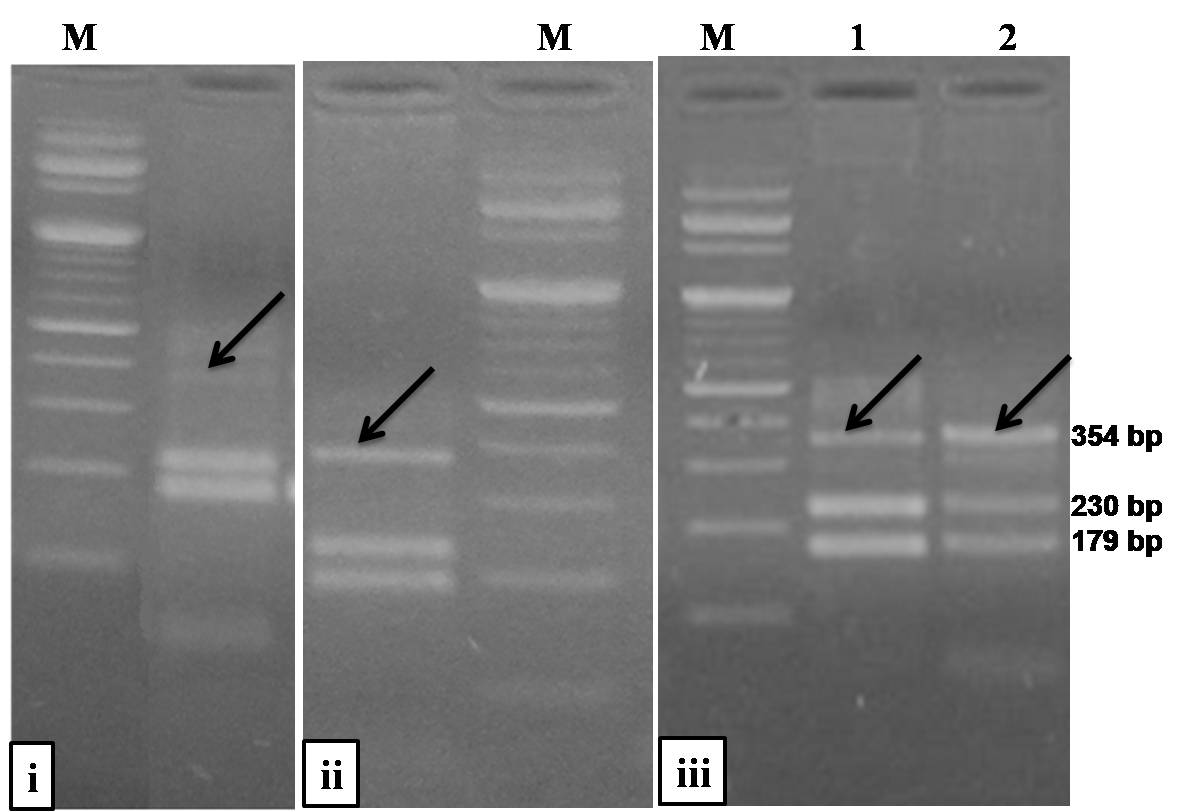


**Fig 3.** Effect of use of 1 and 2 units of SD and Taq polymerase in T-ARMS PCR for genotyping the rs445709131.

**(i);** Genotype pattern generated for rs445709131 heterozygous genotype using 1 unit of SD polymerase

**(ii);** Genotype pattern generated for rs445709131 heterozygous genotype using 2 unit of SD polymerase

**(iii);** Genotype pattern generated for rs445709131 heterozygous genotype using 2 (Lane1) and 1 (Lane 2) unit of Taq DNA polymerase

Arrow indicates the position of expected 354bp band. M (100 bp Molecular marker)

While 1unit of SD polymerase generated low-intense outer amplicons, use of 2 units of SD polymerase gave good amplification of both outer and inner amplicons (Suppl Fig 3i, 3ii). In the case of Taq Polymerase, increasing the quantity of enzyme from 1unit to 2 unit didn’t show any beneficial effect in T-ARMS PCR genotyping (SupplFig 3iii).
